# Insecticide-treated net utilization and associated factors among pregnant women in East Africa: evidence from the recent national demographic and health surveys, 2011–2022

**DOI:** 10.1186/s12936-023-04779-w

**Published:** 2023-11-14

**Authors:** Bewuketu Terefe, Adane Habtie, Bogale Chekole

**Affiliations:** 1 Department of Community Health Nursing, School of Nursing, College of Medicine and Health Sciences, University of Gondar, Gondar, Ethiopia; 2 Department of Public Health, College of Medicine and Health Sciences, Wolkite University, Wolkite, Ethiopia; 3 Department of Comprehensive Nursing, College of Medicine and Health Sciences, Wolkite University, Wolkite, Ethiopia

**Keywords:** Insecticide-treated net, Pregnant women, East Africa

## Abstract

**Background:**

The pregnant woman, the fetus, and the newborn child are all at risk from malaria infection in sub-Saharan Africa. Employing insecticide-treated mosquito nets (ITNs) is one of the most efficient methods for avoiding malaria among expectant mothers. However, there is no literature that describes ITN use among pregnant women in East Africa or the contributing factors. Therefore, this study sought to identify the factors affecting pregnant women’s ITN utilization in East Africa.

**Methods:**

The most recent DHS (Demographic and Health Survey) data for the 11 East African countries from 2011 to 2022 was used. 13,729 pregnant women were examined. To identify factors associated with ITN use, a binary and multiple logistic regression model was built. Variables having a p-value of less than or equal to 0.2 in the binary logistic regression analysis were taken into consideration for the multivariable analysis. In the multiple logistic regression analysis, the adjusted Odds Ratio (aOR) with the 95% Confidence Interval (CI) was provided to proclaim the statistical significance and degree of correlation.

**Results:**

The survey found that just 47.05% (95% CI 46.21, 47.88) of pregnant mothers reported using ITNs. The highest and lowest values were seen in Uganda (64.13%) and Zimbabwe (6.08%). Women age 25-34y (aOR = 1.19; 95% CI 1.11, 1.29), 35–49y (aOR = 1.26; 95% CI 1.13, 1.41) as compared to 15–24 years, poorer (aOR = 1.15; 95% CI 1.04–1.27), middle (aOR = 1.21; 95% CI 1.09, 1.35), and rich (aOR = 1.18; 95% CI 1.06, 1.31) wealth indexes as compared to poorest, having > 5 family size (AOR = 0.84; 95% CI 0.78, 0.91) primary (aOR = 1.49; 95% CI 1.36, 1.65), and secondary/higher education (aOR = 1.52; 95% CI 1.35, 1.70) as compared to not educated, and married women (aOR = 1.64; 95% CI 1.44, 1.86) have shown a statistically significant association with ITN utilization among pregnant women.

**Conclusion:**

With a variety of risk variables, including age, wealth, family size, and education, pregnant women in East Africa rarely use ITNs. There is a need to create and strengthen malaria prevention programmes, especially among pregnant women who do not use ITNs, based on the variables mentioned.

## Background

Malaria is a disease that may be prevented and treated, yet it nevertheless has a terrible effect on people’s health and way of life all over the world, especially in tropical nations [1, 2]. Pregnancy-related malaria is a recognized medical emergency that must be treated in a health facility setting to avoid future negative effects, such as maternal fatalities [3, 4]. The World Health Organization (WHO) estimated that there were more than 241 million cases of malaria and 0.6 million fatalities worldwide in 2021 [5]. Additionally, between 24 and 40% of expectant mothers globally were exposed to malaria during pregnancy [5, 6]. According to the recent WHO report, the highest prevalence of malaria exposure during pregnancy was 40.7% in West Africa, followed by 39.8% in Central Africa, and 20% in East and Southern Africa [5]. Compared to non-pregnant women, pregnant women have a threefold increased chance of developing severe malaria and a twofold increased risk of death [3]. Infants under five and pregnant women are among the group of people who have a notably higher chance of suffering from a serious illness [5, 7].

Anaemia, intrauterine growth restriction (IUGR), and other issues like low birth weight, preterm delivery, and trans placental parasitaemia are commonly linked to malaria [8–10]. For instance, in malaria-endemic areas, 20% of all low-birthweight newborns and one-fourth of all cases of severe maternal anaemia are brought on by malaria [7]. However, 13.3 million (33%) of the predicted 40 million pregnancies in the 38 African nations with moderate to high transmission in 2021 were affected by malaria.

The use of insecticide-treated bed nets (ITN), intermittent preventive therapy with sulfadoxine–pyrimethamine (IPTp-SP), and early case detection are methods for preventing malaria during pregnancy, according to a WHO guideline [11–13]. As a result, it is advised that all expecting mothers sleep under an ITN as early as possible in their pregnancy—ideally, before being pregnant. The pregnant woman and her fetus will be protected against malaria if an ITN is given at the initial point of contact [13, 14]. According to studies, 28 million pregnant women in sub-Saharan Africa do not obtain ITN services, despite the fact that the majority of the continent government accepted the WHO standards for malaria prevention [5, 14–16].

Several reports show that the use of ITNs is extremely low among pregnant women (PW) and children under the age of five (UC5) in sub-Saharan Africa [17, 18]. Poor ITN utilization is a result of various sociodemographic related factors, low levels of knowledge and awareness, as well as ITN-related factors (such as ITN accessibility, adequacy, quality, physical condition, maintenance, replacement, and efficacy) [19–22]. These elements, however, could change over time and in different environments.

This study sought to evaluate the use of ITN among pregnant women in the area using recent nationally representative data. The results of this study will give policy makers, implementers, and scholars updated information about the proportion of pregnant women who use ITNs in the region.

## Methods

### Study setting and period

Demographic and health survey data from 2011 to 2022 secondary analysis of data from the East African nations of Burundi, Kenya, Comoros, Madagascar, Malawi, Mozambique, Rwanda, Tanzania, Uganda, Zambia, and Zimbabwe, was performed using the most recent demographic and health survey (DHS).

### Data source

The demography and health survey (DHS) program’s official database (www.measuredhs.com), was used to obtain the data. Population, health, and nutrition monitoring and impact evaluation indicators can be used using data from demographic and health surveys, which are nationally representative household surveys. The DHS employs a stratified two-stage cluster design, with enumeration areas (EA) being the first stage and a sample of homes being picked from each EA in the second. Comprehensive survey methodology [23]. Person record (PR) databases were employed in each nation for this investigation. All pregnant women aged 15 to 49 who were chosen and interviewed for the use of ITN on the previous night in each nation provided the data. Sampling weights are correction factors that are added to each case in tabulations to account for variations in cases’ probabilities of being chosen and interviewed for an interview within a sample, whether by design or accident. To increase the number of cases available (and hence decrease sample variability) for specific locations or subgroups for whom data are required, the sample in DHS surveys is typically picked with unequal probability. In this instance, weightings must be used when statistics are tabulated in order to provide the appropriate representation. Corrections for varying response rates are also made when weightings are determined as a result of sample design. In this study a weighted sample of 13,729 pregnant women from each of the 11 East African nations whose DHS surveys we used were included in the study (Table 1).

**Table 1:** Countries, sample size, and survey year of demographic and health surveys included in the analysis for 11 East African countries

Country	Survey year	Sample size (weighted)	Percentage (weighted)
Burundi	2016/17	1401	10.20
Kenya	2022	1728	12.59
Comoros	2012	348	2.53
Madagascar	2021	1264	9.21
Malawi	2016/17	1885	13.73
Mozambique	2011	1451	10.56
Rwanda	2019/20	871	6.34
Tanzania	2015/16	11,221	8.17
Uganda	2016	1858	13.53
Zambia	2018	1165	8.49
Zimbabwe	2015	638	4.64

### Study population

Women who were expecting at the time of the survey were the target demographic. All women who regularly resided in the chosen houses as well as those who stayed the night before the survey were eligible to participate in the interview process. In the study’s final analysis, a weighted sample of 13,729 pregnant women was used.

### Variables of the study

Outcome variable: the outcome variable of the study was percentage of pregnant women age 15–49 who slept the night before the survey under an insecticide-treated net (ITN). A factory-treated net that does not need any additional treatment is known as an insecticide-treated net (ITN). This is also referred to as a long-lasting insecticidal net (LLIN). If the pregnant women reported sleeping with an ITN, the response variable was coded as “Yes” = 1 unless it was “No” = 0, respectively. Any missing data was examined in accordance with the DHS statistics guide [24].

### Independent variables

The independent variables were the ages of the women (15–24, 25–34, 35–49), their educational level (no education, primary, secondary/higher), their working status (working, not working), their wealth index (poorest, poorer, middle, rich), the sex of the household head (male, female), the household size (less than or equal to five, six and more), the current marital status of women (never in a union, married, widowed/divorced/separated), number of under five children (one, two, and more than two), having at least one types mass media exposure either watching to television, listening to radio, or reading to magazines or book (yes/no), person who usually decides on respondent’s health (respondent herself, together with husband/partner, husband alone, other relatives), distance from health facility (a big problem/not a big problem), husband education (not educated, primary, secondary or higher), husband working status (currently working/not currently working), visited by health field work (yes/no), visited health facilities in the last 12 months (yes/no), parity (first, second or third, fourth and above), and residence types (urban/rural),

### Data management and statistical analysis

Stata version 17 is used to extract, recode, and analyse data. Weighting was used throughout the study to ensure representativeness and non-response rate, as well as to obtain a suitable statistical estimate (robust standard error) [25]. The intra-class correlation (ICC) coefficient was used to analyse the data because it could be hierarchical; however, because it was only about 2.5%, it did not fulfill the minimal requirement to be used in the study. Thus, it was shown that traditional logistic regression was more effective than the multilevel model.

Variables with a p-value of 0.2 or below in the bivariable analysis were considered for the multivariable analysis. The multivariable logistic model was used to identify the characteristics that are related to ITN usage among pregnant women and produced the adjusted Odds Ratio (aOR) with 95% Confidence Interval (CI) that best fits the model. Descriptive metrics, such as frequency count and proportion for categorical data, were utilized to summarize the descriptive data. Bivariable logistic regression was used to choose potential variables for multiple logistic regression. A logistic model was fitted to test for multicollinearity among the independent variables using the variance inflation factor. The overall fitness of the final regression model was further assessed using the Hosmer and Lemeshow test. The statistical significance threshold for the final model was set at p 0.05.

## Results

### Sociodemographic characteristics of the study participants

This survey comprised weighted samples of pregnant women from 13,729 in East Africa. In terms of age of the women, approximately 6163 (44.89%) of them were from 15 to 24 years old range. Similarly, about 10,854 (79.05%) of them were male household heads. In terms of educational level, slighter more than half 7069 (51.48%) of the participants had enrolled in primary education. Regarding household wealth index about 5198 (36.86%), of them were found in the rich wealth quantile. Furthermore, more than half 10,252 (74.67%), and 8861 (64.54%) of the households have only one under five children, and less than six family members, respectively. About 9125 (66.46%) of women have at least one type of mass media exposure (either watching television, listening to radio or reading books/magazines). The majority of women, 11,682 (85.09%) and 10,368 (75.51%), were married and came from rural areas, respectively. Similarly, majority 9,458 (68.78%) of women have more than 3rd parity. Only 959 (12.53%) of them can make a decision to their own healthcare access (Table 2).

### Factors associated with ITN utilization among east African pregnant women

The odds of ITN use was (AOR = 1.19; 95% CI 1.11, 1.29), and (AOR = 1.26; 95% CI 1.13, 1.41) times higher among pregnant women aged from 25 to 34, and 35 to 49 as compared to those whose age is from 15 to 24 years old, respectively. Similarly, those women from poorer, middle, and rich households had shown 1.15 (AOR = 1.15; 95% CI 1.04, 1.27), 1.21 (AOR = 1.21; 95% CI 1.09 1.35), and 1.18 (AOR = 1.18; 95% CI 1.06, 1.31) times higher odds of ITN use, respectively, as compared to those from poorest households. Women from household size of above five had 16% (AOR = 0.84; 95% CI 0.78, 0.91) lower odds of ITN use as compared to those households with below six household size. Women who had completed their primary, and secondary/higher educational levels have shown 1.49 (AOR = 1.49; 95% CI 1.36, 1.65), and 1.52 (AOR = 1.52;95% CI 1.35, 1.70) times to utilize ITN compared to uneducated pregnant women, respectively. As compared to never married women, those married women have shown 1.64 (AOR = 1.64; 95% CI 1.44, 1.86) more times to utilize ITN during their pregnancy period (Table 3).

**Table 2:** Socio-demographic and maternal related characteristics of respondent’s on ITN utilization among pregnant women in East African countries (weighted n = 13,729, and unweighted 13,699)

Variables	Weighted sample	Frequency
Age of the respondent		
15–24	6163	44.89
25–34	5587	40.69
35–49	1980	14.42
Sex of the household head		
Male	10,854	79.05
Female	2876	20.95
Wealth index		
Poorest	3014	21.95
Poorer	2905	21.16
Middle	2613	19.03
Rich	5198	36.86
Family size		
≤ 5	8861	64.54
> 5	4869	35.46
Umber of under five children		
One	10,252	74.67
Two	2756	20.07
More than two	722	5.26
Education		
Not educated	2307	16.80
Primary	7069	51.48
Secondary/higher	4354	31.71
Mass media exposure		
No	9125	66.46
Yes	4605	33.54
Working status		
No	3217	23.43
Yes	10,513	76.57
Distance to facility		
No problem	9049	65.91
Big problem	4681	34.09
Husband working		
No	361	2.63
Yes	13,369	97.37
Husband education (n = 7655)		
Not educated	2897	37.85
Primary	3636	47.49
Secondary and higher	1122	14.65
Visited health facility		
No	3712	27.03
Yes	10,018	72.97
Visited by health worker		
No	13,203	96.16
Yes	527	3.84
Person who usually decides on respondent’s healthcare (n = 7655)		
The woman	959	12.53
Woman and husband	4572	59.73
Husband alone	2113	27.61
Other relatives	11	0.14
Parity		
First	1457	10.61
2nd or 3rd	2815	20.50
> 3rd	9458	68.88
Marital status		
Never married	1234	8.98
Married	11,682	85.09
Divorced/widowed/separated	814	5.93
Residence		
Urban	3362	24.49
Rural	10,368	75.51

**Table 3:** Factors associated with ITN utilization among pregnant women in east Africa: based on the 2011–2022 DHS data

ITN utilization	No, n (%)	Yes, n (%)	COR (95% CI)	Std. errs.	z	AOR (95% CI)	P > z
Dependent variables							
Age							
15–24	3434 (55.73)	2729 (44.27)	1			1	
25–34	2840 (50.84)	2747 (49.16)	1.20 (1.11, 1.29)	0.046	4.55	**1.19 (1.11, 1.29)**	0.0001
35–49	996 (50.29)	984 (49.71)	1.16 (1.05, 1.28)	0.069	4.15	**1.26 (1.13, 1.41)**	0.0001
Wealth index							
Poorest	1720 (57.07)	1294 (42.93)	1			1	
Poorer	1544 (53.14)	1361 (46.86)	1.20 (1.08, 1.33)	0.061	2.62	**1.15 (1.04, 1.27)**	0.009
Middle	1345 (51.46)	1268 (48.54)	1.29 (1.16, 1.43)	0.066	3.55	**1.21 (1.09, 1.35)**	0.0001
Rich	2662 (51.21)	2536 (48.79)	1.27 (1.163, 1.39)	0.064	3.06	**1.18 (1.06, 1.31)**	0.002
Family size							
≤ 5	4548 (51.33)	4313 (48.67)	1			1	
> 5	2722 (55.91)	2147 (44.09)	0.80 (0.74,0.85)	0.031	− 4.65	**0.84 (0.78, 0.91)**	0.0001
Education							
Not educated	1386 (60.08)	921 (39.92)	1			1	
Primary	3634 (51.41)	3434 (48.59)	1.47 (1.34, 1.61)	0.073	8.17	**1.49 (1.36, 1.65)**	0.0001
Secondary/higher	2250 (51.67)	2104 (48.33)	1.49 (1.35, 1.64)	0.088	7.20	**1.52 (1.35, 1.70)**	0.0001
Mass media exposure							
No	4847 (53.12)	4278 (46.88)	1			1	
Yes	2423 (52.63)	2182 (47.37)	1.03 (0.96, 1.11)	0.038	0.57	1.02 (0.95, 1.10)	0.570
Working status							
No	1682 (52.27)	1536 (47.73)	1			1	
Yes	5589 (53.16)	4924 (46.84)	0.97 (0.90, 1.05)	0.041	− 1.13	0.95 (0.88, 1.04)	0.259
Distance to facility							
No problem	4785 (52.88)	4264 (47.12)	1			1	
Big problem	2486 (53.10)	2195 (46.90)	1.02 (0.95, 1.09)	0.037	0.07	1.01 (0.93, 1.08)	0.945
Husband working							
No	198 (54.84)	163 (45.16)	1			1	
Yes	7072 (52.90)	6297 (47.10)	1.01 (0.82, 1.25)	0.114	0.27	1.03 (0.83, 1.28)	0.784
Visited health facility							
No	2002 (53.93)	1710 (46.07)	1			1	
Yes	5268 (52.59)	4750 (47.41)	1.06 (0.99, 1.15)	0.043	1.40	1.06 (0.98, 1.15)	0.163
Visited by health worker							
No	7007 (53.07)	6196 (46.93)	1			1	
Yes	263 (49.89)	264 (50.11)	1.06 (0.88, 1.26)	0.098	0.44	1.04 (0.87, 1.25)	0.662
Parity							
First	774 (53.10)	683 (46.90)	1			1	
2nd or 3rd	1496 (53.13)	1319 (46.87)	0.99 (0.87, 1.13)	0.066	− 0.06	0.99 (0.88, 1.13)	0.950
4th and above	5001 (52.88)	4457 (47.12)	0.98 (0.87, 1.09)	0.057	− 0.16	0.99 (0.88, 1.11)	0.872
Marital status							
Never married	803 (65.06)	431 (34.94)	1			1	
Married	5945 (50.89)	5737 (49.11)	1.74 (1.54, 1.97)	0.109	7.41	**1.64 (1.44, 1.86)**	0.0001
Divorced/widowed/separated	523 (64.20)	291 (35.80)	1.02 (0.84, 1.22)	0.096	− 0.25	0.98 (0.81, 1.18)	0.800
Residence							
Urban	1837 (54.64)	1525 (45.36)	1			1	
Rural	5433 (52.40)	4935 (47.60)	0.99 (0.92, 1.07)	0.051	1.85	1.09 (0.99, 1.20)	0.064

Bold values are significant at a P-value of <0.05 in the final model

### Prevalence of ITN utilization among pregnant women

**Fig. 1:**
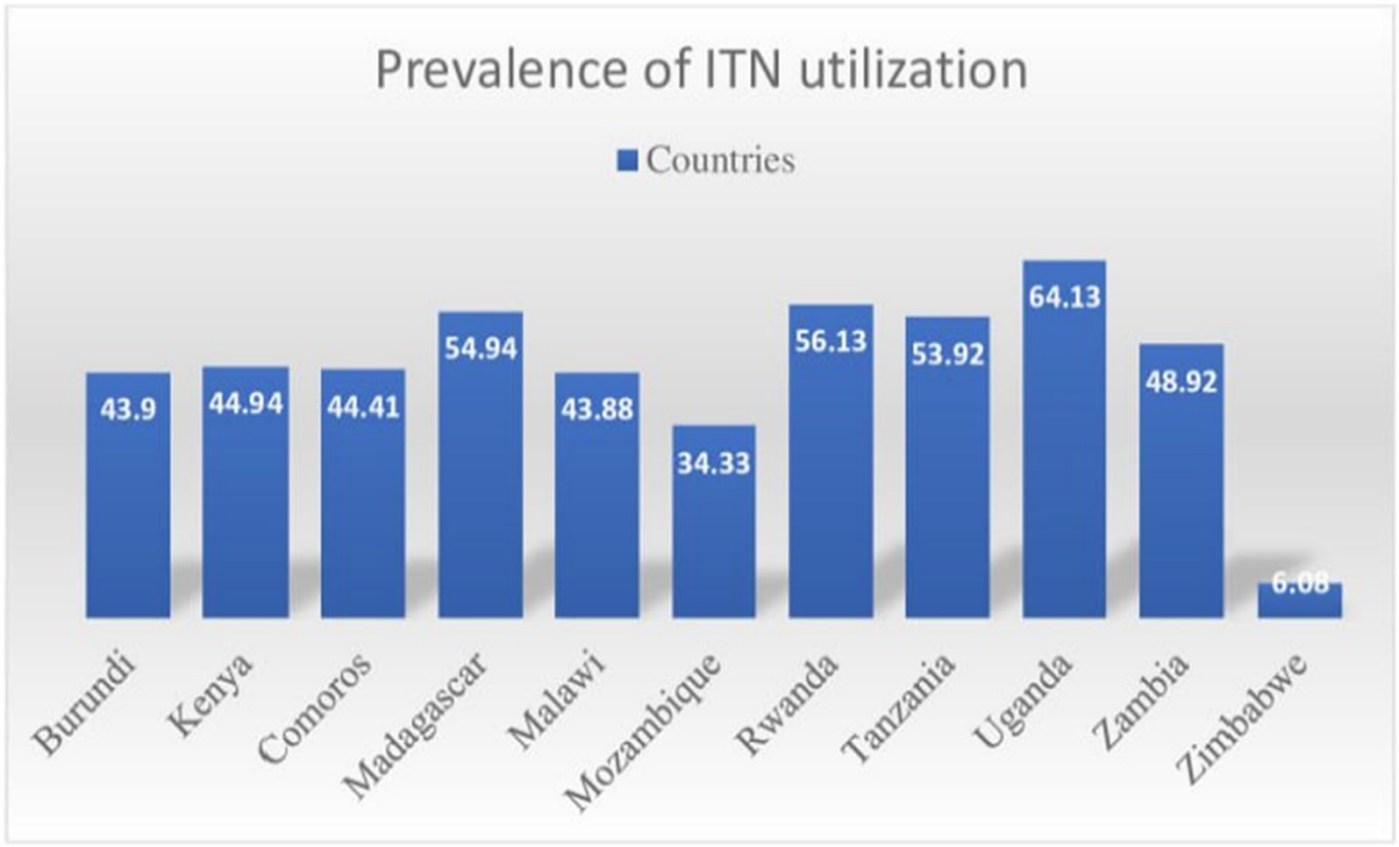
The prevalence of ITN utilization across countries among pregnant women in East Africa from 2011 to 2022

The overall utilization of ITN by pregnant women in East Africa was about 47.05% (95% CI 46.21, 47.88). Uganda, and Zimbabwe have shown the highest and lowest prevalence of 64.13%, and 6.08% ITN utilization among pregnant women, respectively (Fig. 1).

## Discussion

The current study was done to analyse the prevalence and parameters related with ITN usage among pregnant women in East Africa to give crucial information about this group because there has not been any research regarding ITNs utilization among pregnant women in East Africa. The survey found that just 47.05% of expectant mothers used ITN. This utilization is higher than that of some other malaria-endemic nations like Uganda (35%) [26], and Ethiopia (39.9%) [27], however, it is also lower than that of other nations like the Democratic Republic of the Congo (71.4%) [28], Mozambique (68.4%) [29], and Ghana (61%) [30]. However, a logical argument would be that different countries have varied malaria management tactics since they have diverse malaria risk levels due to varying climatic and geographic conditions. Additionally, the time of year during which data was gathered may be a factor, since more people use mosquito nets when malaria transmission is at its highest, which typically coincides with wet/rainy seasons [31]. This emphasizes how important it is to maintain bed net availability, early procurement, and education/sensitization on mosquito bed net usage. Prior studies, and this study descriptive results have shown that access remains a major impediment to the use of mosquito bed nets; consequently, a readily available supply greatly enhances their use [32, 33].

Regarding maternal age, this study found that older pregnant women were more likely to use ITN than younger women. These results concur with earlier research works [34–36], which may be because older women are more likely to have had previous pregnancies, lived experiences with pregnancy, or even negative effects of malaria, better communication, financially independent, better healthy behaviour with society, and positive attitudes, making them more likely to grasp the significance of using mosquito bed nets.

Additionally, this study revealed that married women had higher likelihood of using ITN than women who had never been married. Substantial prior literatures from Rwanda, Ghana, and Nigeria have revealed similar association regarding marital status, and ITN utilization among pregnant women [34, 37, 38]. The support of spouses or partners, desired pregnancy, improved ANC follow-ups, and community involvement are all wonderful opportunities for married women to develop their interpersonal skills and knowledge [39, 40].

It is observed that the use of ITN had a favourable correlation with a higher household wealth index. In Nigeria, pregnant women’s use of ITN has previously been linked positively to the household wealth index [34, 41, 42]. Wealthier individuals have a better chance of healthcare access, and education [43]. Although ITNs are typically given away free of charge to expectant mothers, purchasing ITNs may occasionally be necessary owing to ITN shortages, and the poorest people may be neglected [43]. The process of improving Universal Health Coverage is still ongoing [44]. Poorest and struggling rural households work incredibly hard to make a living wage so they can pay for healthcare and their daily needs.

The study found a negative correlation between using ITN and having a big family size (more than five). This could have a number of causes, including an imbalance between family size and the number of ITN, bed rooms, or separate sleeping areas. As shown by the study from Uganda and Ethiopia, using ITNs in single-room homes could be difficult due to a lack of convenient space that does not permit mosquito net hanging [45, 46]. It is advised to make ITN consistently available, to provide ITN in accordance with their quantity, and to encourage their use among these huge family sizes.

The results of this study also show that respondents’ educational status had an impact on how they used ITN. Primary-level and higher educated respondents were 1.49 and 1.52 times more likely to use ITN than respondents without a formal education enrollment, respectively. Higher educational status respondents were more likely to use ITN than respondents with little formal education, according to other research conducted in Cameroon [47], Nigeria [48], sub-Saharan Africa [49], and Uganda [50]. The association may exist because educated mothers find it simple to read and comprehend information about ITN and malaria. In addition, educated mothers may seek medical attention and consult books, periodicals, and newsletters. In addition, educated mothers may also experience improved empowerment and financial security. Consequently, the impact of transportation costs, cultural barriers, and male dominance may be negligible among educated women.

The findings apply to all pregnant women in the area since we used a weighted dataset from the most recent national DHS. The study offers insightful data as a first look at ITN use and its contributing factor among this unique group in the East African region. Additionally, DHS uses higher sample sizes and standardized high-quality data gathering techniques, which allows to compare the results to those of other nations. The usage of ITNs is socially desirable, and the majority of the data were self-reported with no record justification, thus there is a danger of information and recollection bias. The accuracy of the estimate of women’s ITN use may have been impacted by this. Due to the inconsistent timeliness of the data, this study examined and contrasted different countries without taking temporal variation into account as an independent variable. This might have an impact on the study’s findings. Therefore, care should be taken when applying this study’s conclusions. A more thorough comparison of the observed correlates with other nations/regions was constrained by the absence of precise information on malaria prevention and control in other countries. Additionally, because ITN usage was only evaluated the night before the survey, it might not fully reflect the usage pattern over time.

## Conclusions

The percentage of East African pregnant women utilizing ITNs is regarded as low by the WHO and the sustainable development goals (SDGs) plans. This study investigated the association between individual and societal characteristics and the use of ITN among pregnant women in East Africa using the most recent DHS records. The age of the women, wealth position, household size, married status, and educational attainment were characteristics in the final model that were linked to ITN use. Therefore, it is suggested to spread information about the malaria prevention programme more widely through the media, considering community campaigns and ITN distribution based on family size. Additionally, households with low levels of income should be given special consideration, and additional research should be conducted by taking important factors like knowledge and attitude, perception, and other culturally pertinent characteristics into account.

## Data Availability

All data concerning this study are accommodated and presented in this document. The detailed data set can be freely accessible from the www.dhsprogram.comwebsite.
